# Serglycin Is Implicated in the Promotion of Aggressive Phenotype of Breast Cancer Cells

**DOI:** 10.1371/journal.pone.0078157

**Published:** 2013-10-31

**Authors:** Angeliki Korpetinou, Spyros S. Skandalis, Aristidis Moustakas, Kaisa E. Happonen, Heidi Tveit, Kristian Prydz, Vassiliki T. Labropoulou, Efstathia Giannopoulou, Haralambos P. Kalofonos, Anna M. Blom, Nikos K. Karamanos, Achilleas D. Theocharis

**Affiliations:** 1 Laboratory of Biochemistry, Department of Chemistry, University of Patras, Patras, Greece; 2 Ludwig Institute for Cancer Research, Science for Life Laboratory, Uppsala University, Uppsala, Sweden; 3 Department of Laboratory Medicine, Division of Medical Protein Chemistry, Lund University, Malmö, Sweden; 4 Department of Molecular Biosciences, University of Oslo, Oslo, Norway; 5 Karamandaneion Childrens’ Hospital, Patras, Greece; 6 Clinical Oncology Laboratory, Division of Oncology, University Hospital of Patras, Patras Medical School, Patras, Greece; University of Insubria, Italy

## Abstract

Serglycin is a proteoglycan expressed by some malignant cells. It promotes metastasis and protects some tumor cells from complement system attack. In the present study, we show for the first time the *in situ* expression of serglycin by breast cancer cells by immunohistochemistry in patients’ material. Moreover, we demonstrate high expression and constitutive secretion of serglycin in the aggressive MDA-MB-231 breast cancer cell line. Serglycin exhibited a strong cytoplasmic staining in these cells, observable at the cell periphery in a thread of filaments near the cell membrane, but also in filopodia-like structures. Serglycin was purified from conditioned medium of MDA-MB-231 cells, and represented the major proteoglycan secreted by these cells, having a molecular size of ∼250 kDa and carrying chondroitin sulfate side chains, mainly composed of 4-sulfated (∼87%), 6-sulfated (∼10%) and non-sulfated (∼3%) disaccharides. Purified serglycin inhibited early steps of both the classical and the lectin pathways of complement by binding to C1q and mannose-binding lectin. Stable expression of serglycin in less aggressive MCF-7 breast cancer cells induced their proliferation, anchorage-independent growth, migration and invasion. Interestingly, over-expression of serglycin lacking the glycosaminoglycan attachment sites failed to promote these cellular functions, suggesting that glycanation of serglycin is a pre-requisite for its oncogenic properties. Our findings suggest that serglycin promotes a more aggressive cancer cell phenotype and may protect breast cancer cells from complement attack supporting their survival and expansion.

## Introduction

Serglycin is a proteoglycan (PG) with a 17 kDa core protein containing a characteristic domain rich in serine/glycine repeats, which serves as the attachment site for up to eight glycosaminoglycan (GAG) chains [1]. Although serglycin does not contain a transmembrane domain, this PG was initially discovered at the cell membrane of rat L2 yolk sac tumor cells [2] and was the first PG gene to be cloned [3]. Serglycin is mainly expressed by cells of hematopoietic origin and is located in secretory granules or vesicles. It carries either chondroitin sulfate (CS), dermatan sulfate (DS) or heparin/heparan sulfate (HS) chains depending on cell-type. The biological function of serglycin is not fully elucidated. However, results obtained with serglycin knockout mice suggest that serglycin may play a role in the delivery of proteins into secretory granules and/or directing the secretion of these molecules [4], [5]. Serglycin is co-localized with tissue-type plasminogen activator [6] and chemokine growth-related oncogene α (GROα/CXCL1) [7] in endothelial cells. and regulates the expression of matrix metalloproteinase 9 (MMP9) and urokinase plasminogen activator (uPA) in Madin-Darby canine kidney cells [8]. Serglycin is constitutively secreted by multiple myeloma cells [9] and aggressive nasopharygeal cancer cells [10]. Elevated expression of serglycin promotes aggressiveness of nasopharygeal cancer cells and correlates with the formation of distant metastases [10]. Cell surface associated serglycin promotes the adhesion of myeloma cells to collagen type I and up-regulates the biosynthesis of matrix metalloproteinases [11]. It has been shown that serglycin forms stable heteromers with proMMP9 *in vitro* modulating the properties of the enzyme [12]. Serglycin inhibits the classical and the lectin pathways of the complement system, thus protecting myeloma cells from complement attack [13]. Complement is activated through three different routes [14]. The classical pathway is activated by the formation of antibody-antigen complexes and their recognition by the first complement component C1. The lectin pathway is triggered when mannose-binding lectin (MBL) or ficolins bind to polysaccharide molecules present on the surface of microorganisms. The alternative pathway is initiated by properdin or by autoactivation of the complement component C3 and its deposition on surfaces of activating pathogens. All three pathways merge at the level of the C3 convertase and have a common terminal pathway, which leads to the deposition of the membrane attack complex (MAC) and the lysis of the target cell [14]. Complement activation is often associated with the deposition of complement proteins on tumor cell surfaces, indicating that complement is activated in the tumor tissue or in its vicinity. Therefore, complement effectors generated through this process might contribute to the immune surveillance of malignant cells [15], [16].

Breast carcinoma is considered to be one of the main causes of cancer mortality and several studies have demonstrated abnormal expression of PGs in breast cancer [17]. Breast cancer cells express cell-surface associated PGs such as syndecans [17], and the matrix PGs versican and decorin, which are mainly synthesized by stromal cells, are accumulated in the tumor stroma [18]. The abnormal expression of such molecules contributes to breast cancer biology.

Although the expression of PGs in breast cancer has been extensively studied, there are no published data on the expression of serglycin. In this study, we show that serglycin is highly expressed in breast cancer tissues and by an aggressive breast cancer cell line. Serglycin secreted by aggressive breast cancer cells inhibits both the classical and the lectin pathways of complement by directly binding to C1q and MBL in a similar manner as serglycin secreted by myeloma cells. Overexpression of serglycin promotes breast cancer cell growth, migration and invasion. Our data reveal a novel role of serglycin in breast cancer and the promotion of the disease.

## Materials and Methods

### Ethics Statement

Permission of the local ethical committee of Lund University was obtained for the collection and preparation of normal human serum. Written consent was obtained from all participants (healthy volunteers) who donated blood for preparation of serum. A human tissue microarray (TP483) was purchased from US Biomax Inc (www.biomax.us/). According to Greek laws and regulations no ethical permit is required for use of such commercially available tissue microarrays in research.

### Antibodies, Enzymes, Purified Proteins and DNA Constructs

Goat antiserum to human C1q was purchased from ICN Biomedicals Inc., while goat anti-rabbit horseradish peroxidase (HRP)-conjugated secondary antibody was from Sigma-Aldrich and rabbit anti-goat HRP-conjugated secondary antibody was from Dako Cytomation. Rabbit polyclonal antibody against serglycin was prepared as previously described [9]. Chondroitinases ACII and ABC were purchased from Seikagaku. Serglycin isolated from culture medium of multiple myeloma cell lines was used as standard [9]. C1q was purchased from Sigma-Aldrich. C1-inhibitor (C1inh) was purchased from Complement Technologies, and MBL was from Statens Serum Institute (Denmark). Factor H was purified according to a previously published method [19]. Normal human serum (NHS) was prepared from the blood of 8 healthy volunteers and frozen to −80°C immediately after separation. Serglycin fused to green fluorescent protein (GFP) has previously been described [20], and the construct GFP- serglycin (VSG) in pEGFP-N3 vector (Clontech Laboratories Inc, Mountain View, California, USA) was further used as template for the construction of GFP- serglycin without GAG sites (VSG/−GAG). VSG/−GAG was amplified using primers with inserted MluI restriction sites (underline), forward primer (5′TATACGCGTTTCCTAACGGAAATGGAACAGGATT-3′) flanking 3′end and reverse primer (5′ATCACGCGTGTAGTCCTCAGAAAGTGGGAAGATAC-3′) flanking 5′ end of the GFP-serglycin-GAG domain. The PCR product consisting of GFP-serglycin without the GAG domain and pEGFP-N3 was amplified by using Advantage®2 Polymerase (Clontech), followed by MluI treatment and ligation. The MluI restriction site insert replaced the GAG domain in serglycin with amino acids threonine and arginine.

### Dataset

The expression of serglycin across 51 human mammary cell lines was provided by Neve et al. [21] using Affymetrix HG-U133A arrays. The results on the expression of serglycin were visualized by the Cell Lines module in the Gene Set Analysis using GOBO database (http://co.bmc.lu.se/gobo/gobo.pl).

### Cell Culture and Stable Cell Line

MDA-MB-231, MDA-MB-468 and MCF-7 breast cancer cell lines were purchased from the American Type Culture Collection (ATCC). MDA-MB-468 cells were cultured in RPMI 1640 medium (Biochrom), with 2 mM L-glutamine supplemented with 10 mM HEPES, 1 mM sodium pyruvate, 4.5 g/L glucose, 1.5 g/L sodium bicarbonate and 10% fetal bovine serum (FBS), as recommended by ATCC. MDA-MB-231 and MCF-7 cells were cultured in Eagle’s minimum essential medium with Earle’s BSS and 2 mM L-glutamine (EMEM, Biochrom) and supplemented with 1 mM sodium pyruvate, 0.1 mM nonessential amino acids, 1.5 g/L sodium bicarbonate, 10 µg/mL human insulin (Sigma-Aldrich), 1% Pen/Strep (10000 units/mL penicillin and 10000 units/mLstreptomycin, Biochrom) and 10% FBS, as recommended by ATCC. Cells were cultured at 37°C in 5% CO_2_. Cells were transfected with FuGENE® HD transfection reagent (Roche) with GFP-empty vector (V), GFP-serglycin (VSG) and GFP- serglycin without GAG attachment sites (VSG/−GAG), following the manufacturer’s instructions. For establishment of stable cell lines, 800 µg/mL Geneticin G-418 (Gibco) was added to culture media, changing the media twice a week. Clonal selection was performed after 10 days, keeping the cells under continuous pressure with Geneticin G-418.

### Preparation of Culture Medium Supernatant

One liter of culture supernatant was prepared from MDA-MB-231 cells grown to 80% confluency in culture medium containing serum. After centrifugation, the supernatant was passed through a sterile filter and stored at −20°C until further analysis.

### Quantification of Serglycin Concentration in Culture Medium Supernatants

1×10^6^ cells were plated per 100 mm dishes and cultured at normal culture conditions. After 18 h, cells were starved for 48 h, when cultures reached 80% confluency and culture supernatants were collected, centrifuged at 3000 rpm for 5 min and concentrated with Vivaspin 6 ultrafiltration devices (Sartorius Biotech). Equal amounts of protein measured by Coomassie Plus-Bradford Assay™ Kit (Thermo Scientific), were treated with 0.02 units chondroitinase ABC in 50 mM Tris-HCl, pH 7.5 at 37°C for 2 h. Samples were reduced with β-mercaptoethanol in Laemmli sample buffer and separated by SDS-PAGE. The proteins were transferred to Immobilon-P PVDF membranes (Millipore). The membranes were blocked in 5% non-fat dry milk in PBS-0.1% Tween-20 for 2 h and were then incubated with 0.55 µg/mL rabbit polyclonal anti-serglycin overnight at 4°C. Membranes were incubated for 1 h at room temperature with peroxidase-conjugated secondary goat anti-rabbit antibody. Detection of the immunoreactive proteins was performed by chemiluminescence horseradish peroxidase Pierce® ECL Western blotting substrate, according to the manufacturer’s instructions. Serglycin contents were analyzed through Western blotting using increasing amounts of standard serglycin isolated from myeloma cells [9] and expressed as protein to create a standard curve each time. Both culture supernatants and standard serglycin were treated with 0.02 units chondroitinase ABC as above. Protein band density quantification was calculated using Scion Image software.

### Isolation of PGs by DEAE-Sephacel and Sepharose CL-4B

Culture supernatants were brought to a final concentration of 10 M formamide, 50 mM sodium acetate buffer, pH 6.3 (column buffer), containing protease inhibitors (10 mM EDTA-Na_2_, 10 mM benzamidine hydrochloride, 1 mM phenylmethylsulfonyl fluoride). Each medium was passed through a DEAE-Sephacel (Sigma-Aldrich) column (40-ml bed volume) equilibrated with the above formamide-sodium acetate buffer. The column was washed with 3 bed volumes of the above buffer containing 0.2 M NaCl to elute the non-proteoglycan components, and PGs were fractionated by gradient elution ranging from 0.2 M to 1.0 M NaCl. Fractions were collected, and aliquots were precipitated in 80% ethanol and 1.3% potassium acetate. Precipitates were dissolved in deionized-distilled water and the GAG contents were quantified by the dimethylmethylene blue method [22]. Aliquots of fractions were also precipitated and subjected to SDS-PAGE analysis using a 4% stacking - 10% separating gel. Gels were stained with 0.1% toluidine blue in 0.1 M acetic acid, followed by staining with 0.3% coomassie blue in 40% (v/v) methanol, 10% (v/v) acetic acid. Serglycin was detected throughout the dissolved aliquots with Western blotting analysis as described above. The major PG population eluting with 0.55–0.7 M NaCl was pooled, concentrated with Vivaspin 20 Ultrafiltration devices (Sartorius Biotech) and subjected to gel permeation chromatography on a Sepharose CL-4B (Sigma-Aldrich) column. Elution was done with 4 M guanidine hydrochloride, 50 mM sodium acetate, pH 5.8. Fractions were collected and aliquots precipitated as above. PGs were monitored by the dimethylmethylene blue method and SDS-PAGE analysis, and serglycin was detected with Western blotting analysis after chondroitinase ABC digestion as described above.

### Characterization of the Glycosaminoglycan Moiety of Serglycin by Capillary Electrophoresis

The isolated serglycin was analyzed before and after treatment with chondroitinase ABC and/or chondroitinase AC II, or a mixture of heparitinases. Digestion with chondroitinase ABC was performed in 50 mM Tris-HCl, pH 7.5, at 37°C for 6 h, using 0.02 units/10 µg of hexuronic acid. Chondroitinase ACII digestion was performed in the same buffer at pH 6.0 under the same conditions. Treatment with heparin lyases I, II, and III was performed in 20 mM sodium acetate, pH 7.0, containing 1 µmol of calcium acetate at 37°C overnight. Aliquots of the digests were analyzed by capillary electrophoresis using an HP^3D^CE (Agilent Technologies) as previously described [23]. Separation and analysis were done on an uncoated, fused silica capillary tube (75 µm inner diameter, 55 cm total length, 50 cm effective length to the detector) at 25°C. The capillary tube was washed for 1 min with 0.1 M NaOH before each run, and then with the operating buffer (15 mM orthophosphate, pH 3.0) for 4 min. Samples were introduced by the pressurized mode at the cathode (reverse polarity) and separated at 20 kV. Detection was performed at 232 nm using a diode array detector. Quantification was performed using the HP ChemStation software provided by the manufacturer with the instrument.

### RNA Isolation, cDNA Synthesis and Reverse Transcription PCR

Cells were cultured in 10 cm cell culture dishes up to 80% confluency. Total RNA was extracted using total RNA isolation NucleoSpin RNA II kit (Macherey-Nagel) according to the manufacturer’s instructions. Integrity of RNA was confirmed by electrophoresis on agarose gels stained with GelRed nucleic acid gel stain (Biotium). RNA was quantified by measuring absorbance at 260 nm using infinite M200 (Tecan). First strand cDNA was synthesized by PrimeScript 1st strand cDNA Synthesis kit (TaKaRa) according to the manufacturer’s instructions. For those reactions, 2 µg of RNA were used in a total volume of 20 µl. In order to compare serglycin gene expression within the three breast cancer cell lines, semi-quantitative reverse transcription – polymerase chain reaction (RT-PCR) was performed with DyNAzyme II DNA Polymerase kit (Finnzymes). Serglycin transcript levels were quantified against β-actin transcript levels, using gene-specific primers (serglycin forward: 5′ AATGCAGTCGGCTTGTCCTG-3′ and serglycin reverse: 5′-TGGTGTCAAGGTGGGAAAAT-3′ resulting in amplicon size of 483 bp. The analysis of the serglycin gene inserted into the pEGFP-N3 vector used forward: 5′ TGCAAACTGCCTTGAAGAAA-3′ and reverse: 5′-ATCCATGTTGACCCAAGTCC-3′ resulting in amplicon size of 316 bp for intact serglycin gene and 268 bp for serglycin lacking GAG attachment sites. β-actin was analyzed with forward: 5′- GTGGGGCGCCCCAGGCACCA-3′ and reverse: 5′- CTCCTTAATGTCACGCACGATTTC-3′ resulting in amplicon size of 539 bp. For PCR reaction, samples containing 125 ng of cDNA were amplified in a total volume of 50 µL [10 mM Tris-HCl pH 8.8, 50 mM KCl, 1.5 mM MgCl_2_, 0.1% Triton X-100, containing dNTP mix (each at 0.2 mM), both downstream and upstream primers (each at 200 nM), and 1 unit of DyNAzyme™ II DNA polymerase]. The PCR amplification was done at: 95°C for 20 sec, annealing temperature 51°C for 20 sec, 72°C for 30 sec (MiniCycler™, MJ Research). Equal volumes of the PCR products were electrophoresed on 1% agarose gels stained with GelRed nucleic acid gel stain (Biotium), and the results were analyzed with band density quantification, using Scion Image software.

### Proliferation Assay

Stably transfected cells were harvested with trypsin, re-suspended in PBS and counted. Cells were plated in triplicates in 24 well plates (1.5×10^4^ cells per well) and incubated for several time points under normal culture conditions. 3-(4,5-dimethylthiazol-2-yl)-2,5-diphenyltetrazolium bromide (MTT) was added to the wells to a final concentration of 1 mM, and cells were incubated for 2 h at 37°C. This assay is based on the measurement of metabolic activity and the produced formazan was diluted with 100 µL of 0.33% (v/v) HCl in isopropanol per well and absorbance was measured at 570 nm. Standard curves of cell numbers related to absorbance were made for each cell line using an increasing number of cells plated per well. Four hours after plating, MTT was added and absorbance was measured as described above.

### Migration and Invasion Assays

Stably transfected cells (5×10^5^) were plated in triplicates in 12 well plates. Cells were incubated until confluency (approximately for 24 h) with normal culture conditions. Wounds were made using a sterile pipette tip, debris was removed, fresh culture medium was added, cells were monitored and images were captured at various time points (0, 24 and 48 h). Wound area was determined at various time intervals using Image J software.

To evaluate migratory properties of stably transfected cells, 1×10^5^ cells were suspended in culture medium supplemented with 0,5% FBS and loaded onto the top of Transwell chambers (24-well plate) equipped with 8.0 µm pore-size polycarbonate membranes (Corning). For invasion assay, 1×10^5^ cells were suspended in culture medium supplemented with 0,5% FBS and loaded onto the top of Transwell chambers (24-well plate) equipped with matrigel-coated cell culture inserts (BD Biosciences). As chemotactic stimuli in the bottom chambers was used culture medium supplemented with 10% FBS. After 48 h (migration assay) or 72 h (invasion assay) of incubation, cells on the upper surface of the filter were mechanically removed with a cotton swab, and those which migrated underneath the surface were fixed and stained with Giemsa dye. Photographs were taken using an Olympus microscope (CKX 41) and the number of cells was counted.

### Colony Formation Assay

For the colony formation assay, a 0.6% low-melting-temperature agarose (Chembiotin), base layer was prepared in EMEM supplemented with 10% FBS and Pen/Strep. A 0.3% low-melting-temperature agarose top layer was prepared in EMEM supplemented with 10% FBS, 100 U/mL of penicillin, 100 µg/mL of streptomycin and 10 µg/mL human insulin containing 5×10^4^ cells. After 12 days of incubation under normal culture conditions, cells were stained with 0.05% crystal violet. Photographs from six random fields were taken, and cell colonies were counted using Image J software.

### Immunofluoresence

Breast cancer cells were cultured in chamber slides until they reached 70% confluency (Nunc, Rochester, USA). In some cases a linear wound was made, debris was removed, fresh culture medium was added, and cells were further incubated for 24 h at 37°C. Cells were then fixed with 4% paraformaldehyde in PBS for 30 min at room temperature and subsequently rinsed with PBS. Permeation of cell membranes was accomplished by a 10 min incubation of the cells with 0.1% (v/v) Triton X-100 in PBS at room temperature. Cells were washed with PBS and incubated with 3% BSA solution supplemented with 10% FBS for 1 h at room temperature. Cells were then rinsed twice with PBS, and incubated overnight at 4°C with a polyclonal rabbit anti-serglycin antibody (1.66 µg/mL) diluted in blocking solution. All above treatments were performed under normal light to quench endogenous GFP signals. Cells were rinsed with PBS and then incubated with an anti-rabbit antibody conjugated with Alexa Fluor 488 (1∶1000, Invitrogen, Molecular Probes, Eugene, Oregon, USA) diluted in blocking solution for 1 h at room temperature in the dark. Cells were rinsed with PBS and then incubated with phalloidin-TRITC in blocking solution (10 nM, Sigma-Aldrich) for 30 min at room temperature in the dark. After rinsing with PBS, a fluorescent dye was used for nucleus staining (1 mg/mL bisBenzimide, H 33342 Sigma-Aldrich). Cells were mounted on glass slides. Examination of staining was performed with a Leica TCS NT confocal laser-scanning microscope equipped with an ArKr laser. Non-permeabilized cells and cells treated only with secondary antibody were also stained as controls. For each experiment the excitation and sensitivity of the detector were adjusted so that the corresponding negative controls gave barely detectable signals. Subsequently all settings were kept equal for the analysis.

### Immunohistochemistry

Reactivity to the rabbit anti-serglycin antibody was also studied using sections of routinely fixed (4% buffered formalin) and paraffin-embedded breast cancer tissues. For this purpose, a human tissue microarray (TP483) was purchased from US Biomax Inc (www.biomax.us/) (Rockville, MD, USA), containing among other 2 normal breast tissues and 8 breast carcinomas. Sections (5 µm) were deparaffinized, rehydrated, and subjected to epitope retrieval in a microwave oven using 7.14 mM sodium citrate pH 6.0. Endogenous peroxidase quenching was performed with incubation in TBS containing 0.9% H_2_O_2_ for 20 min in the dark. Blocking was performed with 2% BSA in TBS, and sections were incubated with 1.38 µg/mL rabbit anti-serglycin in antibody diluent (Dako). The staining to demonstrate serglycin was performed using the Dako REAL™ EnVision™ detection system (peroxidase/DAB+, rabbit/mouse). The sections were counterstained with hematoxylin.

### Hemolytic Assay

To measure the activity of the classical complement pathway, sheep erythrocytes (Swedish National Veterinary Institute) were coated with amboceptor (Dade Behring). Increasing amounts of serglycin expressed in terms of GAG content isolated from MDA-MB-231 breast cancer cells were incubated with NHS diluted in DGVB^2+^ buffer (2.5 mM veronal buffer pH 7.4, 70 mM NaCl, 140 mM glucose, 0.1% gelatine, 1 mM MgCl_2_ and 0.15 mM CaCl_2_) for 15 min on ice in microtiter plates. Approximately 5×10^8^ cells/mL of antibody-sensitized erythrocytes were added to the NHS-serglycin mixtures, and the plates were incubated for 1 h at 37°C with shaking. Hemolysis was measured as the absorbance of the supernatant at 405 nm after centrifugation for 5 min at 2000 rpm.

To measure the activity of the alternative pathway, rabbit erythrocytes were used, and NHS was diluted in Mg^++^EGTA buffer (2.5 mM veronal buffer, containing 70 mM NaCl, 140 mM glucose, 0.1% gelatin, 7 mM MgCl_2_, 10 mM EGTA, pH 7.4) to prevent activation of the classical pathway. Serum pre-incubated with serglycin isolated from myeloma cells or factor H served as positive controls for the classical and alternative pathway assays, respectively.

### Inhibition of Complement Deposition

Microtiter plates (Maxisorp, Nunc) were coated with 100 µg/ml mannan in 75 mM Na-carbonate buffer pH 9.6 at +4°C over night. The plates were blocked with 1% BSA in PBS to prevent unspecific binding. NHS diluted to a concentration of 0.8% in GVB^2+^ (5 mM veronal buffer pH 7.4, 144 mM NaCl, 1 mM MgCl_2_, 0.15 mM CaCl_2_, and 1% gelatin) was pre-incubated for 15 min on ice with increasing amounts of serglycin expressed in terms of GAG content after which it was added to the plate. As a negative control, BSA was incubated with serum whereas D(+)mannose was used as positive control. The plates were incubated at +37°C for 30 min after which deposition of complement was detected with antibodies against C3b (A0063, Dako) and C4b (Q0369 Dako) followed by HRP-conjugated secondary antibodies (P0399, Dako). The plates were developed with o-phenylenediamine (OPD) substrate (Dako) and H_2_O_2_ and the absorbance at 490 nm was measured (Cary 50 MPR microplate reader, Varian).

### Solid Phase Binding Assay

Microtiter plates were coated with 5 µg/ml serglycin measured as GAG content or 1% BSA diluted in 75 mM Na-carbonate buffer pH 9.6 at +4°C over night after which they were blocked with 1% BSA in PBS. MBL and C1q diluted in 50 mM Hepes pH 7.4, 150 mM NaCl, 2 mM CaCl_2_ were added to the plate at increasing concentration after which bound MBL and C1q were detected with a monoclonal antibody (HYB 131-01B; Bioporto) and a rabbit polyclonal antibody (55115 ICN Biomedicals), respectively. HRP-conjugated anti-mouse and anti-rabbit antibodies were added and the plates were developed as described above. Data were analyzed according to the modified equation ΔA/[S] = ΔAmax/K_D_ - ΔA/K_D_, where ΔA is the specific absorbance measured in a given concentration of C1q or MBL, [S] is the given concentration, ΔAmax is the specific absorbance at the saturation and K_D_ the dissociation constant for the binding between C1q or MBL and the immobilized molecule. The data have then been plotted using a Scatchard-type plot with ΔA/[S] in the y-axis and ΔA in the x-axis. K_D_ values were determined from the slopes of the linear plots.

## Results

### Assessment of Serglycin Expression in Breast Cancer Tissues

**Figure 1:**
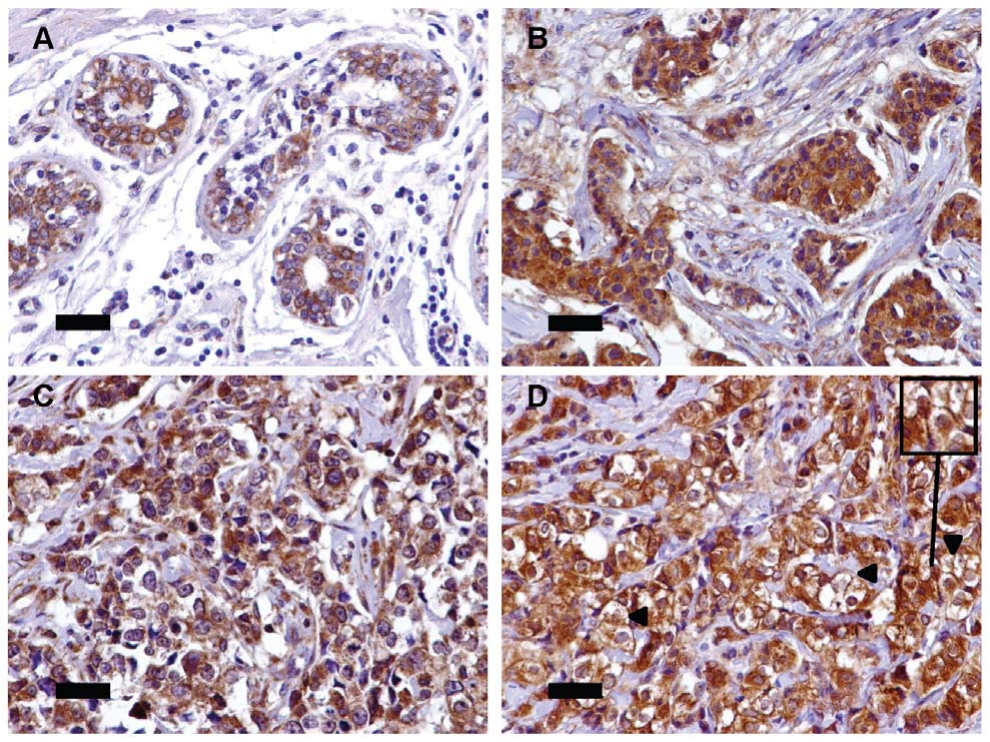
Immunohistochemical reactivity of serglycin in normal breast and breast carcinoma. Normal epithelial breast cells in mammary glands were moderately reactive with the polyclonal antibody against serglycin and the reactivity was cytoplasmic (A). Breast cancer cells were stained strongly for serglycin in grade 2 breast carcinomas (B–D). The serglycin reactivity was mainly cytoplasmic, although in some cases cell-surface associated staining was detected in breast cancer cells (D, arrowheads and insert). Bars, 50 µm.

The expression of serglycin in normal breast tissue and breast carcinoma was examined by immunohistochemistry in paraffin-embedded tissues. Using a polyclonal antibody, serglycin was found to be expressed by normal epithelial cells in mammary glands of healthy tissues (Fig. 1A) showing a moderate cytoplasmic staining. Notably, serglycin was ubiquitously synthesized by breast cancer cells, in all cases showing a strong cytoplasmic staining (Fig. 1B–D). In some cases, cell membrane-associated reactivity for serglycin was also apparent (Fig. 1D, arrowheads).

### Serglycin is Highly Expressed by Aggressive Breast Cancer Cell Lines

**Figure 2:**
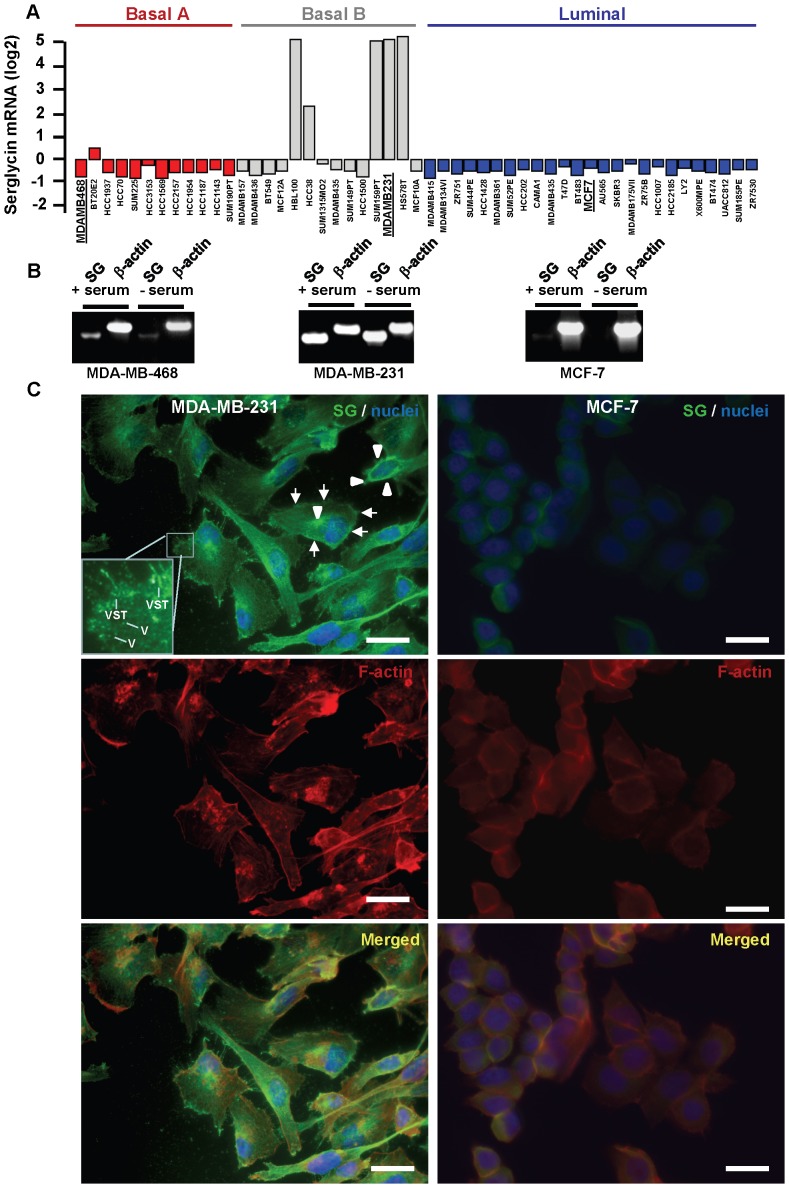
Serglycin is highly expressed in aggressive breast cancer cell lines. (A) Expression of serglycin across the 51 individual breast cancer cell lines grouped in the basal A (red), basal B (grey) and luminal (blue) subgroups. (B) Detection of serglycin gene transcripts in low aggressive MCF-7 (luminal), medium aggressive MDA-MB-468 (basal A) and high aggressive MDA-MB-231 (basal B) breast cancer cells (bold and underlined in figure 2A) cultured in the presence or absence of serum by RT-PCR. The PCR products were analyzed on 1% agarose gels stained with GelRed. (C). Subcellular distribution of serglycin in breast cancer cells. Immunofluorescence staining was done for serglycin (green), nuclei (blue) and F-actin (red) in high aggressive MDA-MB-231 and low aggressive MCF-7 breast cancer cells after permeabilization. MCF-7 cells stained faintly for serglycin in the cytoplasm. Serglycin was found in the perinuclear regions and juxtanuclear area typical of the Golgi complex in MDA-MB-231 cells (arrowheads), and in the cytoplasm and at the cell periphery (arrows). Serglycin distribution was observed in vesicular (V) and vesiculotubular structures (VST) in the cytoplasm (insert). Bars, 25 µm.

The expression of serglycin across 51 human breast cancer cell lines [21] clustered into three subgroups (Luminal, Basal A and Basal B) was obtained by the Cell Lines module in the Gene Set Analysis using GOBO database (http://co.bmc.lu.se/gobo/gobo.pl). Serglycin was over-expressed by breast cancer cells clustered into Basal B subgroup (Fig. 2A). Breast cancer cells that belong to Basal B appeared less differentiated, had a more mesenchymal-like appearance and were highly invasive, as compared to cells clustered into Basal A and Luminal subgroups [21]. To confirm these data in our cell lines, we examined the expression of serglycin in breast cancer cell lines that clustered into three subgroups (MCF-7, MDA-MB-468 and MDA-MB-231 cells, clustered to Luminal, Basal A and Basal B, respectively). Total RNAs from MDA-MB-231 (high aggressive), MDA-MB-468 (medium aggressive) and MCF-7 (low aggressive) breast cancer cells cultured in serum-free medium or in complete culture medium were reverse transcribed and amplified using specific primers for serglycin (Fig. 2B). It was found that high aggressive MDA-MB-231 cells expressed much higher levels of mRNA coding for serglycin compared to medium aggressive MDA-MB-468 cells (Fig. 2B), whereas low aggressive MCF-7 cells hardly expressed detectable amounts of mRNA for serglycin (Fig. 2B). In all cases, the cells cultured in complete medium showed a tendency to higher expression of the serglycin gene compared to cells cultured in serum-free culture medium, although this increase was not statistically significant (not shown).

### Localization of Serglycin in Breast Cancer Cells

MDA-MB-231 and MCF-7 breast cancer cells were permeabilized and stained for serglycin and F-actin. In MDA-MB-231 cells, serglycin displayed a strong perinuclear and juxtanuclear distribution that is typical of the Golgi complex (Fig. 2C). Strong perinuclear staining for serglycin that originated from Golgi complex was also found in endothelial cells [7]. In addition, direct localization of serglycin in Golgi complex was demonstrated in neutrophils by immunoelectron microscopy [24]. The cytoplasmic staining of serglycin was filamentous but did not resemble actin fibers, although partial co-localization with actin was noticed in perinuclear regions (Fig. 2C). Serglycin was also found at the cell periphery, forming a thread of filaments towards the cell membrane (Fig. 2C) and in vesicular or tubular-shaped bright spots in the cytoplasm (Fig. 2C, inset), suggesting that serglycin localized to the plasma membrane and to vesiculotubular structures in the cytoplasm. In MCF-7 cells, the serglycin signal was weak and partially co-localized with the cortical actin at cell-cell adhesion sites (Fig. 2C).

### Quantification of Serglycin Secreted by Breast Cancer Cells

**Figure 3:**
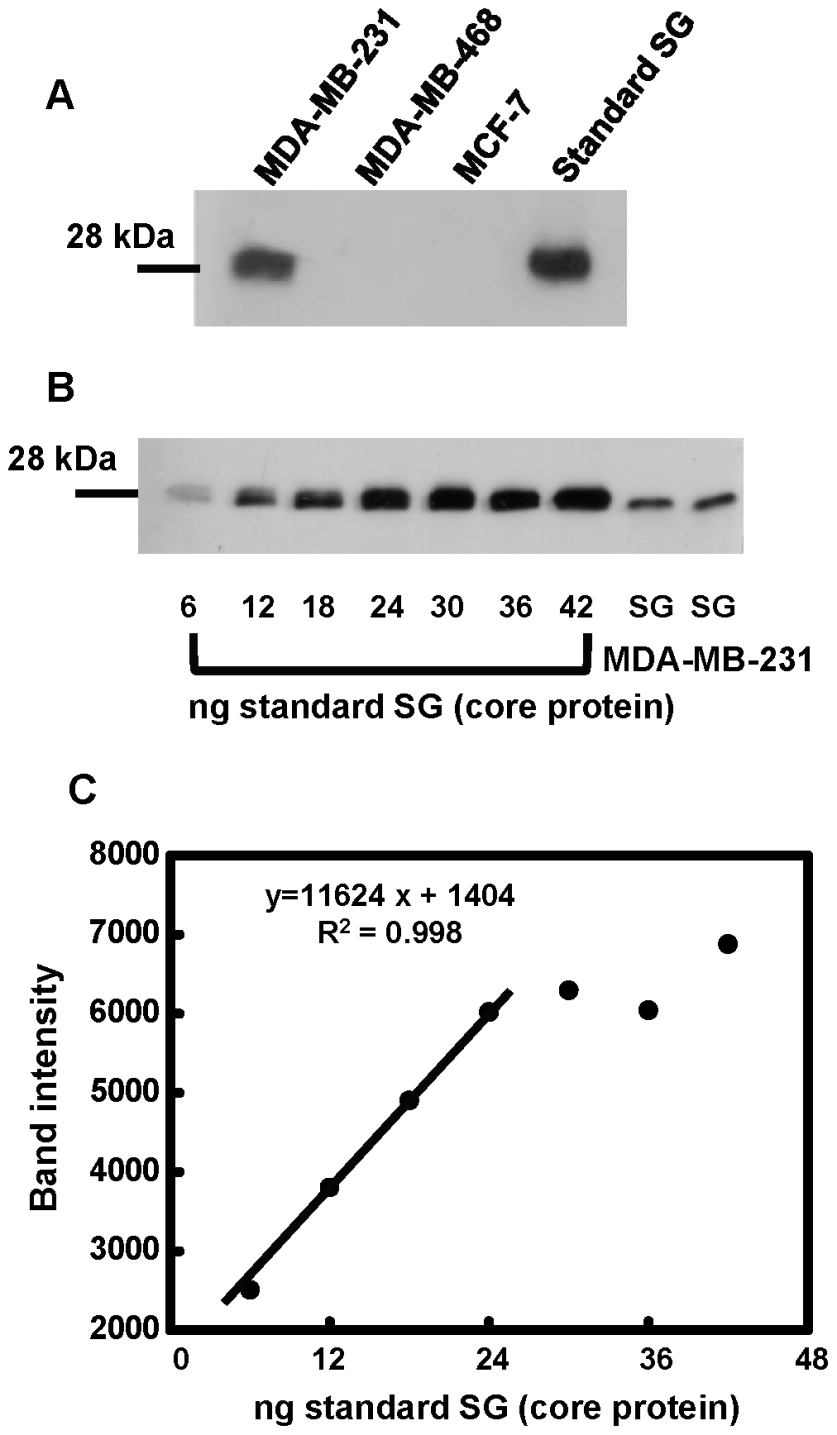
Serglycin is secreted to the culture medium by aggressive breast cancer cells. Equal amounts of protein from concentrated cell culture supernatants of breast cancer cell lines correspond to known number of cells were treated with chondroitinase ABC and subjected to Western blot analysis for serglycin using chondroitinase ABC digested standard serglycin (SG) as positive control (A). (B) Various amounts of standard SG measured as protein from the CAG myeloma cell line and known amounts of protein from concentrated cell culture supernatants from MDA-MB-231 breast cancer cells correspond to known number of cells (two independent experiments) were treated with chondroitinase ABC and were simultaneously analyzed for the presence of SG by Western blotting. (C) A standard curve was created each time by plotting protein band density against the amount of SG subjected to Western blot analysis. A linearity in the standard curve was obtained for amounts up to 24 ng of loaded SG protein (R^2^ = 0.998), and the curve was used for the quantification of SG present in the culture medium of MDA-MB-231 cells.

To evaluate whether serglycin is secreted into the culture medium, breast cancer cells were cultured in serum free medium, and supernatants were collected and concentrated. Equal amounts of protein were digested with chondroitinase ABC and analyzed by Western blotting. Serglycin core protein was only detected in the culture medium of MDA-MB-231 cells (Fig. 3A). We further quantified the serglycin secreted by MDA-MB-231 cells by Western blot analysis using a standard curve with increasing amounts of serglycin (Figs. 3B and C). A linear curve was obtained for serglycin core protein amounts up to 24 ng (Fig. 3C). Using this standard curve, we analyzed serglycin present in the culture medium of MDA-MB-231 cells, and we found that the concentration of serglycin core protein was 44±9 ng/mL corresponding to 44±9 ng serglycin core protein/8.2×10^5^ cells.

### Isolation and Characterization of Serglycin Secreted by MDA-MB-231 Breast Cancer Cells

**Figure 4:**
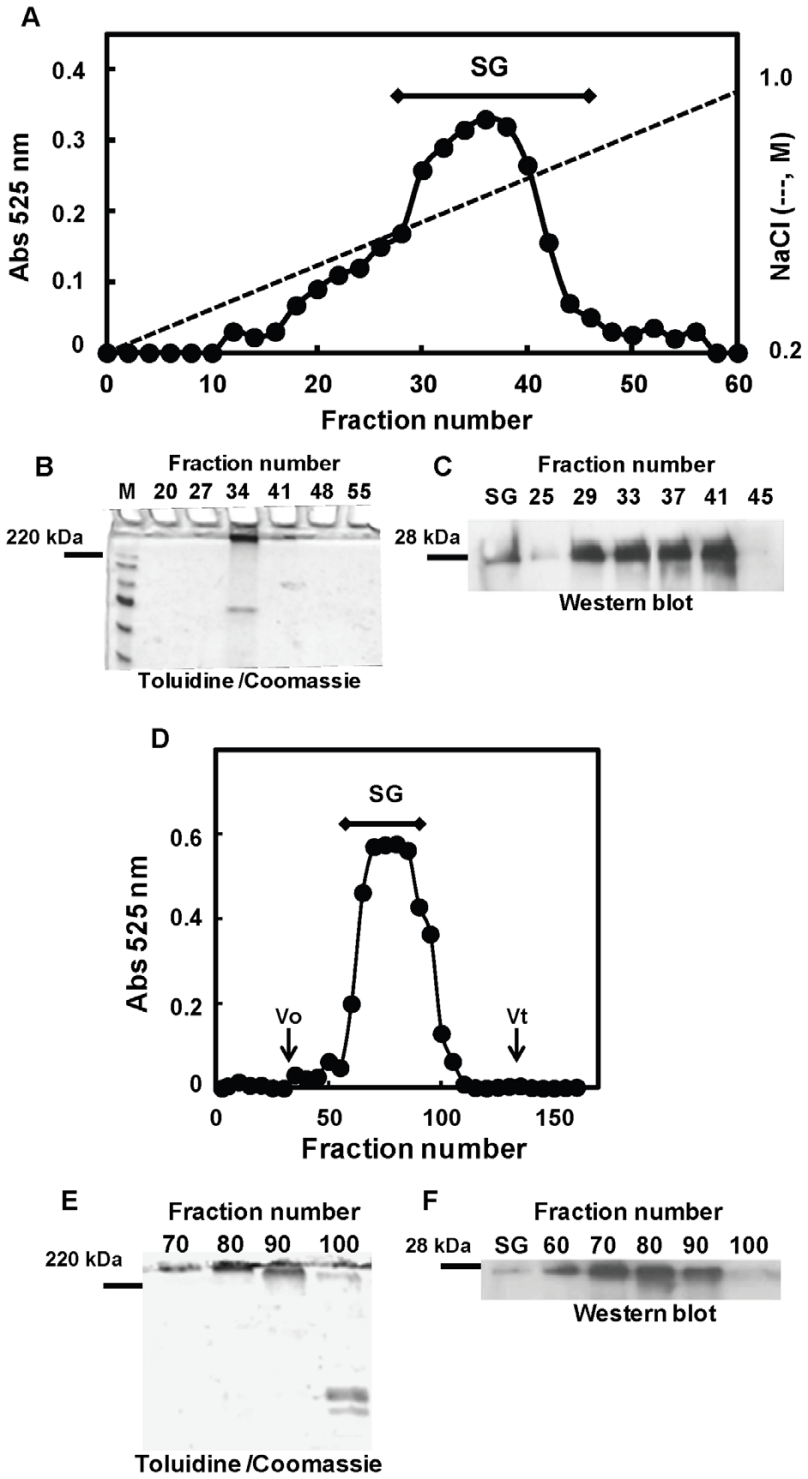
Fractionation of PGs secreted from the MDA-MB-231 breast cancer cell line by anion-exchange and gel permeation chromatographies. One liter of conditioned medium was fractionated on a DEAE-Sephacel column (40 ml bed volume). The column was eluted stepwise with 3 volumes of the formamide buffer described under “Experimental Procedures” containing 0.2 M NaCl and 10 volumes of a NaCl linear gradient ranging from 0.2 to 1.0 M NaCl. Fractions of 6.7 ml were collected, and aliquots were precipitated by the addition of ethanol in the presence of potassium acetate. Precipitates were dissolved in distilled water, analyzed for their GAG content by the DMMB method (A), and subjected to SDS-PAGE analysis using a 4% stacking - 10% separating gel (B). Gels were stained with toluidine blue, followed by staining with coomassie blue. Serglycin was detected throughout the dissolved aliquots by Western blotting analysis after chondroitinase ABC digestion using a polyclonal antibody against serglycin (C) (SG, standard of serglycin used as positive control). A major PG peak was eluted from 0.50 to 0.7 M NaCl and pooled as indicated by the bar. Pooled serglycin was further fractionated by gel permeation chromatography (D). The pooled fractions were chromatographed on a Sepharose CL-4B column. The column was eluted with 4 M guanidine hydrochloride, 50 mM sodium acetate buffer, pH 5.8. Fractions were collected, and PGs were monitored by the DMMB method (D), were subjected to SDS-PAGE following toluidine blue and coomassie staining (E), and were analyzed by Western blotting after chondroitinase ABC digestion (F) (SG, standard of serglycin used as positive control).

Serglycin secreted by MDA-MB-231 cells was isolated and characterized following fractionation of secreted PGs by combined anion-exchange and gel filtration chromatographies. One liter of culture medium was applied on a DEAE-Sephacel column, and PGs were fractionated by linear gradient elution, ranging from 0.2 to 1.0 M NaCl. The major PG population was eluted with 0.5–0.7 M NaCl and was pooled as shown by the bar (Fig. 4A). Aliquots of the fractions were analyzed by SDS-PAGE following toluidine blue and coomassie blue staining (Fig. 4B). The major PG population remained on top of the separating gel with some in the stacking gel, whereas some protein impurities migrated with lower molecular size (Fig. 4B). Aliquots of the fractions were also digested with chondroitinase ABC and subjected to Western blotting (Fig. 4C). Using a specific antibody against serglycin, a core protein of ∼28 kDa was detected, indicating the presence of serglycin in the major PG peak (Fig. 4C). The pooled PGs from DEAE-Sephacel were then analyzed by gel filtration chromatography on a Sepharose CL-4B column (Fig. 4D). The isolated PGs were eluted as a homogeneous population with Kav∼0.5 with an average molecular weight of 300 kDa (Fig. 4D). SDS-PAGE analysis of the column fractions showed the presence of a high molecular mass population, which remained at the top of the separating gel and in the stacking gel. The PG population was well separated from protein impurities that migrated with lower molecular sizes (Fig. 4E). This homogeneous population was characterized by Western blot analysis and was positive for the presence of serglycin with a core protein of ∼28 kDa (Fig. 4F). The absence of other PG species in this population was confirmed following treatment with chondroitinase ABC or a mixture of heparin lyases and Western blotting using specific antibodies for decorin, versican, and syndecans. The amount of isolated serglycin was measured by the dimethylmethylene blue method and whale chondroitin 4-sulfate as GAG standard. The concentration of serglycin was found 0.68 µg GAGs/mL corresponding to 0.68 µg GAGs/8.2×10^5^ cells. Taking into consideration that serglycin GAG content represents about 94.5%, whereas core protein represents about 5.5% of total molecular weight, this GAG amount corresponds to about 40 ng serglycin protein/mL (per 8.2×10^5^ cells) and is in agreement with serglycin concentration in culture medium estimated above. To characterize GAGs on the isolated population, the PGs were digested by chondroitinases ABC and AC II in combination or with chondroitinase AC II alone, and the digestion mixtures were analyzed by capillary electrophoresis. This population carried CS chains, but not DS chains, since treatment with chondroitinase AC II alone or combined digestion with chondroitinases ABC and AC II liberated equal amounts of disaccharides. Furthermore, products corresponding to heparin, heparan sulfate and hyaluronan were not observed, following treatment with the appropriate enzymes and capillary electrophoretic analysis. CS was predominantly constituted of 4-sulfated disaccharides (87%), whereas much smaller amounts of 6-sulfated (10%) and non-sulfated units (3%) were detected.

### Serglycin Inhibits the Classical and the Lectin Complement Pathways

**Figure 5:**
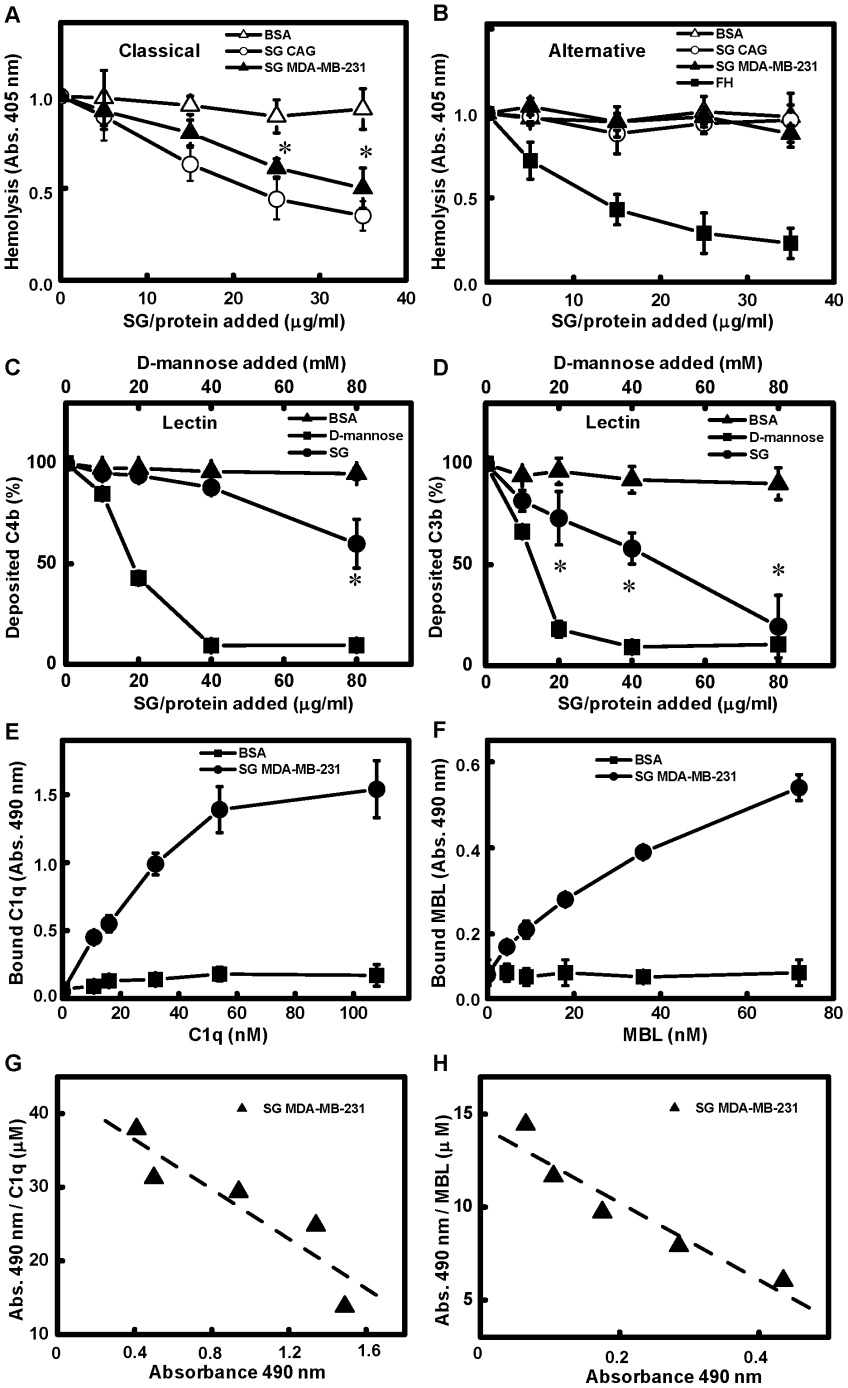
Serglycin inhibits the classical and lectin pathways of complement and binds to complement components C1q and MBL. (A) Serglycin inhibits the classical pathway. NHS was pre-incubated with increasing concentrations of total glycanated serglycin isolated from MDA-MB-231 breast cancer cells and the CAG myeloma cell line (positive control) and mixed with Ab-sensitized sheep erythrocytes. No impact on hemolysis was observed when BSA was used (negative control). Erythrocyte lysis was evaluated after 1 h by measuring the amount of released hemoglobin at 405 nm. (B) Inhibition of the alternative pathway was measured by subjecting rabbit erythrocytes to NHS pre-incubated with serglycin isolated from MDA-MB-231 breast cancer cells and the CAG myeloma cell line and erythrocyte lysis was determined as above. For the alternative pathway, Factor H (FH) and BSA were used as positive and negative controls, respectively. (B and C) To examine the inhibition of the lectin pathway, NHS was pre-incubated with increasing concentrations of total glycanated serglycin isolated from MDA-MB-231 breast cancer cells, D-mannose (positive control) or BSA (negative control) and added to mannan-coated wells. Deposition of C4b (E) and C3b (F) was measured by ELISA. Data were normalized by setting the obtained absorbance for NHS without inhibitor to 1. All data are given as means and SD of three independent assays. Statistical significance of variances compared to negative control was calculated using using a two-way ANOVA test and a Bonferroni post-test (*p<0.05). Wells coated with total glycanated serglycin isolated from MDA-MB-231 breast cancer cells or BSA (negative control) were incubated with increasing concentrations of purified C1q (E) and MBL (F) for 1 h at RT, and bound C1q and MBL were detected with ELISA. Data are given as means and SD of three independent experiments. Scatchard-type plots representing the binding of serglycin isolated from breast cancer to C1q (G) and MBL (H). Data are given as means and SD of three independent experiments.

It has been established that serglycin secreted by myeloma cells inhibits complement [13]. To examine whether serglycin isolated from breast cancer cells exhibits a similar inhibitory profile, we measured the total hemolytic activity of the classical and the alternative complement pathways in the presence of serglycin *in vitro*. When complement activation is induced on the surface of erythrocytes, either by the classical or alternative pathway, complement components deposit onto the cell surface and trigger the formation of a lytic pore. For the classical pathway evaluation, antibody-sensitized sheep erythrocytes were incubated with NHS in the presence of increasing amounts of serglycin and the amount of erythrocyte lysis was determined as a measure of complement activity. Serglycin inhibited the classical pathway of complement in a dose-dependent manner (Fig. 5A). In contrast to the effects seen on the classical pathway, serglycin did not decrease lysis of rabbit erythrocytes induced by activation of the alternative pathway (Fig. 5B). Complement activation and deposition of complement components from serum may also be induced on a synthetic surface coated with a pathway-specific complement activator. To investigate the effect of serglycin on the lectin pathway, we measured the deposition of C3b and C4b on mannan-coated wells in the presence of serglycin. Pre-incubation of NHS with serglycin significantly decreased deposition of C4b (Fig. 5C) and C3b (Fig. 5D). Therefore serglycin inhibits specifically the classical and the lectin pathways without influencing alternative pathway activity, showing a biological activity similar to that of serglycin isolated from multiple myeloma cells [13].

### Serglycin Binds to C1q and MBL

Considering that the inhibitory effect of serglycin isolated from multiple myeloma cells started from the very first steps of the classical and lectin pathways, and this was attributed to the binding of serglycin to C1q in the classical and MBL in the lectin pathway [13], we investigated the binding capacity of serglycin isolated from MDA-MB-231 cells to these complement components. Using a binding assay with immobilized serglycin, we found that C1q (Fig. 5E) and MBL (Fig. 5F) bound serglycin from breast cancer cells dose-dependently. Using Scatchard-type plot (Fig. 5G), we calculated the dissociation constant (K_D_) of the binding between serglycin isolated from breast cancer cells and C1q to be 0.2×10^−^7 M. The affinity of the interaction between breast cancer-derived serglycin and C1q was slightly higher than the affinity between multiple myeloma cell-derived serglycin and C1q (K_D_ = 0.4×10^−^7 M). The latter K_D_-value was calculated from data published before [13] using Scatchard-type plots (not shown).The K_D_ value for the interaction between serglycin from breast cancer cells and MBL was estimated to be 4.8×10^−8^ M (Fig. 5H). In contrast to the affinity to C1q, serglycin from MDA-MB-231 cells showed a lower affinity for MBL than serglycin isolated from the CAG myeloma cell line (K_D_ = 1.9×10^−8^ M). The latter K_D_-value was also calculated from data published before [13] using Scatchard-type plots (not shown).

### Establishment of Stably Transfected MCF-7 Cells Overexpressing Serglycin and Serglycin Lacking GAG Chains

**Figure 6:**
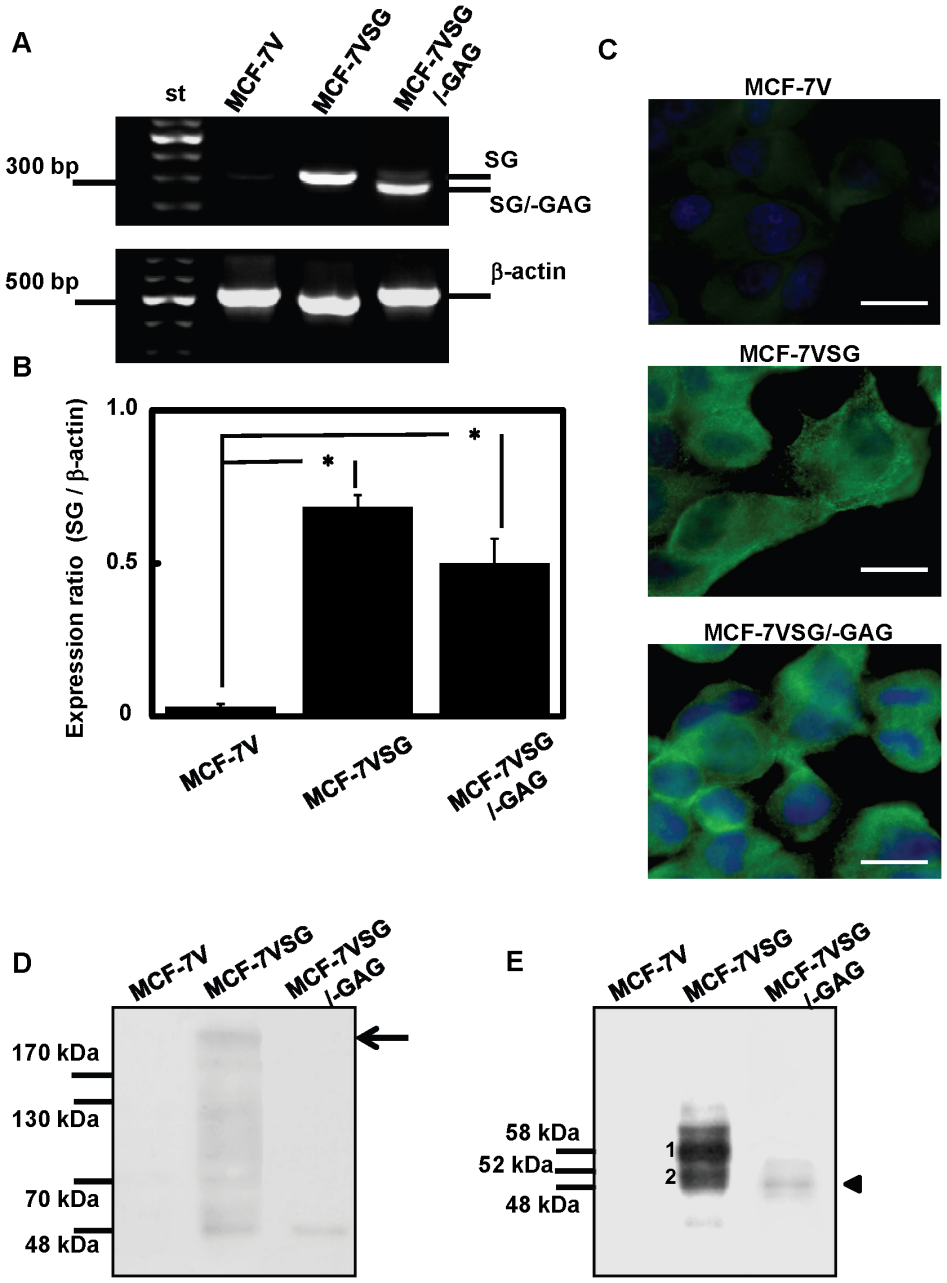
Characterization of serglycin expression in stably transfected MCF-7 cells. Detection of serglycin gene transcripts in MCF-7 breast cancer cells carrying empty vector (MCF-7V), vector with serglycin cDNA (MCF-7VSG) and vector coding serglycin cDNA lacking GAG attachment sites (MCF-7VSG/−GAG) by RT-PCR. The PCR products were analyzed on 1% agarose gels stained with GelRed nucleic acid gel stain (A). The ratio of serglycin (SG) to β-actin was determined following quantification of bands’ densities using Scion Image software (B). Statistical significance of variances was calculated using using a one-way ANOVA test (*p<0.05). (C) Immunofluorescence staining for serglycin (green) and nuclei (blue) in stably transfected MCF-7V, MCF-7VSG and MCF-7VSG/−GAG cells. Cells were cultured in chamber slides, then fixed with 4% paraformaldehyde in PBS, permeabilized with 0.1% Triton X-100 and incubated with a polyclonal rabbit anti-serglycin antibody. Bars, 25 µm. (D) Equal amounts of protein from concentrated cell culture supernatants from stably transfected MCF-7V, MCF-7VSG and MCF-7VSG/−GAG cells correspond to known number of cells were subjected to Western blot analysis using a polyclonal antibody against serglycin without (D) or after treatment with chondroitinase ABC (E). (D) MCF-7VSG secreted to the culture medium a high molecular mass population, which remained on the top of the separating gel and represents highly glycanated serglycin, and low glycanated sub-populations with molecular masses ranging from 170 kDa to 70 kDa. Furthermore, MCF-7VSG and MCF-7VSG/−GAG cells secreted a core protein of 48 kDa that might represent non-glycanated serglycin core containing GFP-tag. (E) When samples were treated with chondroitinase ABC, MCF-7VSG cells found to secrete a major protein core of 58 kDa (1) that might represent the core protein of highly glycanated serglycin containing oligosaccharides remaining after chondroitinase ABC treatment and the GFP-tag. Minor protein cores of 52–48 kDa (2) secreted from MCF-7VSG and a protein core of 48 kDa (arrowhead) secreted from MCF-7VSG/−GAG cells might represent the low glycanated or/and non-glycanated core proteins of serglycin containing the GFP-tag. Arrow shows the top of the separating gel.

MCF-7 cells were transfected with pEGFP-N3 vectors carrying the serglycin cDNA or a truncated serglycin cDNA lacking the serine-glycine repeats onto which GAG chains are attached. Stably transfected cell lines were created, and the expression levels of serglycin were determined by RT-PCR (Fig. 6A). Serglycin mRNA was highly expressed in MCF-7 cells transfected with the plasmid carrying intact serglycin (MCF-7VSG) (14-fold increase) and in cells transfected with the plasmid carrying truncated serglycin (MCF-7VSG/−GAG) (10-fold increase) as compared to cells transfected with empty vector (MCF-7V) (Fig. 6B). Cells were stained for serglycin after quenching the endogenous GFP signal, and the over-expression of serglycin core protein was also confirmed by immunofluorescence microscopy (Fig. 6C). The distribution of transfected serglycin resembled to a large extent the pattern of endogenous serglycin, as analyzed in Fig. 2. We further examined whether serglycin is secreted into the culture medium. Stably transfected cells were cultured in serum free medium, and supernatants were collected and concentrated. Equal amounts of protein were analyzed by Western blotting before and after digestion with chondroitinase ABC (Fig. 6D and E), and quantified as described before using serglycin core protein to obtain a standard curve (not shown). We found that MCF-7VSG secreted to the culture medium differentially glycanated sub-populations of serglycin (Fig. 6D). These cells secreted a highly glycanated population of serglycin that remained on the top of the separating gel and in the stacking gel (not shown), low glycanated sub-populations with molecular masses ranging from 170 kDa to 70 kDa, as well as a non-glycanated core of 48 kDa containing the GFP tag (Fig. 6D). The later non-glycanated core was also detected in the culture medium of MCF-7VSG/−GAG cells (Fig. 6D). The secretion of non-glycanated form of GFP-serglycin, even when the GAG attachment sites are present, has been also demonstrated previously [25]. Samples treated with chondroitinase ABC showed that MCF-7VSG secreted a major protein core of 58 kDa that might represent the core protein of highly glycanated serglycin containing oligosaccharides remaining after chondroitinase ABC treatment and the GFP-tag (Fig. 6E). Minor protein cores of 52–48 kDa secreted from MCF-7VSG and a protein core of 48 kDa secreted from MCF-7VSG/−GAG cells might represent the low glycanated or/and non-glycanated core proteins of serglycin containing the GFP-tag (Fig. 6E). Quantification of the serglycin core proteins showed that MCF-7VSG secreted in high amounts (164 ng of core protein/mL per 8.2×10^5^ cells) the major protein core of 58 kDa (Fig. 6E). Low glycanated or/and non-glycanated protein cores of 52–48 kDa were also secreted in high amounts from MCF-7VSG (102 ng of core protein/mL per 8.2×10^5^ cells) (Fig. 6E). The concentration of the non-glycanated core protein of serglycin (48 kDa) that was secreted from MCF-7VSG/−GAG was 20 ng of core protein/mL per 8.2×10^5^ cells (Fig. 6E).

### Serglycin Affects Cancer Cell Proliferation and Increases Colony Formation

**Figure 7:**
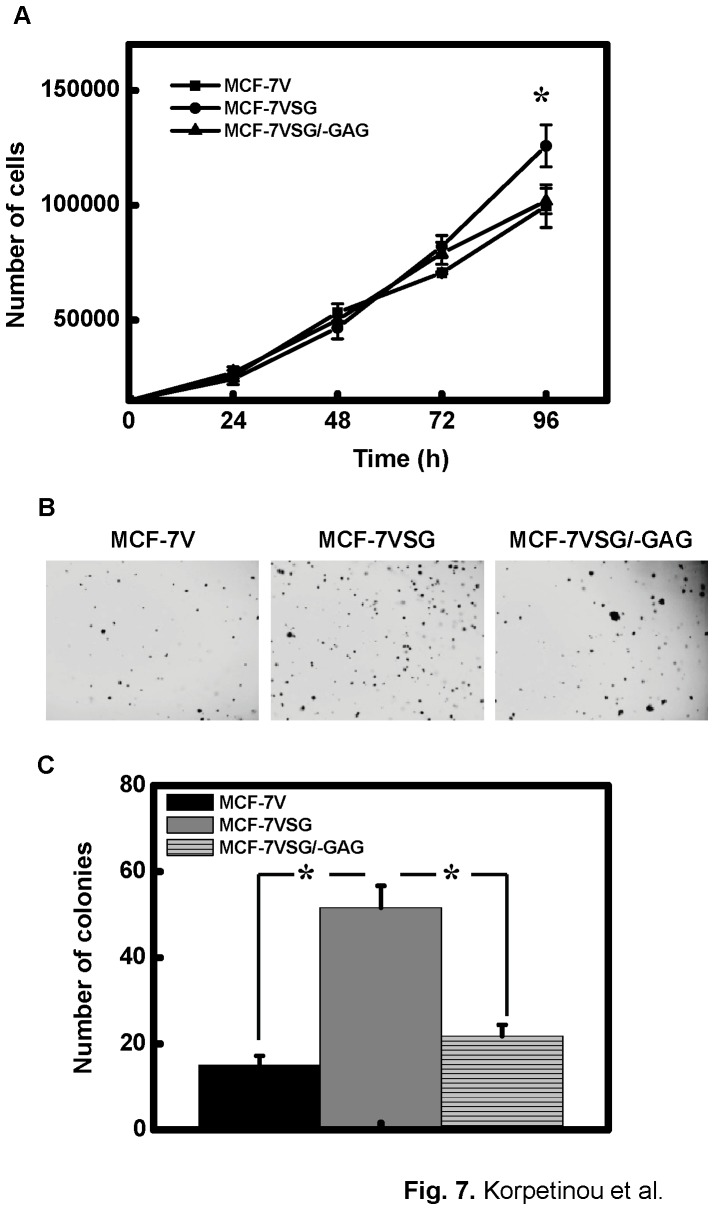
Increased cell proliferation and colony formation in MCF-7 cells over-expressing serglycin. (A) For the evaluation of cell proliferation on adhesive matrix, 1.5×10^4^ of stably transfected cells were plated in triplicates in 24 well plates and incubated for several time points in normal culture conditions. MTT was added in wells and cells were incubated for 2 h at 37°C. The absorbance was measured at 570 nm and related to the number of cells by using a standard curve. Data are given as means and SD of three independent experiments. Statistical significance of variances was calculated using using a one-way ANOVA test (*p<0.05). (B) For the colony formation assay, 5×10^4^ cells were cultured on agarose for 12 days and then stained with 0.05% crystal violet and photographed. (C) Photographs from six random fields were taken, and cell colonies were counted using Image J software. Data are given as means and SD of three independent experiments. Statistical significance of variances between MCF-7VSG and MCF-7V or MCF-7VSG/−GAG cells was calculated using a one-way ANOVA test (*p<0.05).

We examined the role of serglycin in breast cancer cell proliferation, using MCF-7 cells stably over-expressing intact serglycin (MCF-VSG) and non-glycanated serglycin (MCF-7VSG/−GAG). We found that over-expression of intact serglycin slightly increased breast cancer cell growth after long term (96 h) culturing, as compared to mock-transfected (MCF-7V), or MCF-7 cells transfected with serglycin lacking GAG attachment sites (MCF-7VSG/−GAG) (Fig. 7A). Then, we investigated whether serglycin over-expression could influence cell proliferation in a non-adhesive matrix by performing colony formation assays. Cells were cultured for 12 days on agarose, and the colonies formed were stained and counted. As shown in Figure 7B and C, over-expression of intact serglycin significantly increased the number of colonies, compared to mock-transfected cells and cells transfected with non-glycanated serglycin. Cells transfected with serglycin lacking GAG attachment sites showed a tendency to form larger colonies, although the total number of colonies was significantly lower than that of MCF-7VSG, and only slightly higher than that of mock-transfected cells (Fig. 7B and C). These results suggested that serglycin has a role in cell proliferation, since it clearly promoted anchorage-independent growth and that the CS chains of serglycin were required for the promotion of breast cancer cell growth on the non-adhesive matrix (Fig. 7A and C).

### Serglycin Enhances the Migration and Invasion of Cancer Cells

**Figure 8:**
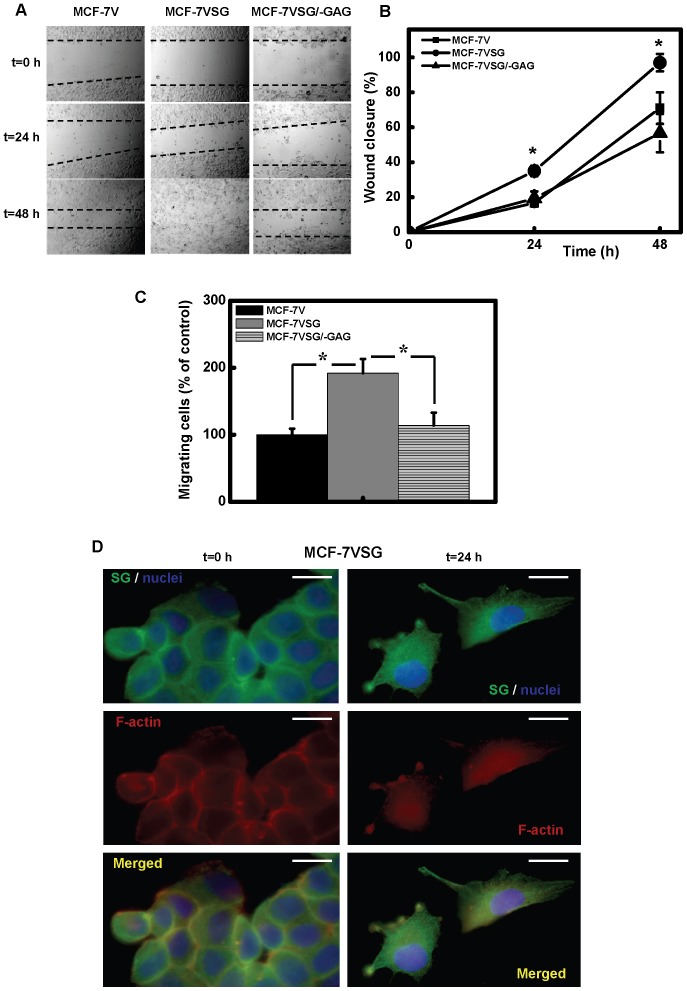
Over-expression of glycanated serglycin increases breast cancer cell migration. Cells (5×10^5^) were plated in triplicates in 12 well plates and cultured until confluence. Then wounds were made using a sterile pipette tip, debris was removed and fresh culture medium was added. The cells were monitored at 0, 24 and 48 h and were photographed (A). Wound areas were quantified at various time intervals using Image J software (B). Data are given as means and SD of three independent experiments. Statistical significance of variances was calculated using a one-way ANOVA test. Asterisk (*) indicates statistically significant differences (*p*<0.05). (C) Migratory properties of the cells were also evaluated by Transwell migration assay. 1×10^5^ cells were suspended in culture medium supplemented with 0.5% FBS and loaded onto the top of Transwell chambers. Cells were then maintained in Transwell chambers for 48 h with 10% FBS as chemotactic stimuli in the bottom chamber. Transmigrating cells were stained with Giemsa and counted. Data are given as means and SD of three independent experiments. Statistical significance of variances was calculated using a one-way ANOVA test. Asterisk (*) indicates statistically significant differences (*p*<0.05). (D) In a set of experiments, cells were cultured on glass coverslips. After wounding with a sterile pipette tip, debris was removed, and cells were either immediately fixed in 3% formaldehyde in PBS (t = 0 h) or cultured for 24 h and then fixed. Immunofluorescence staining for serglycin (green), nuclei (blue) and F-actin (red) in MCF-7VSG cells was performed. Bars, 25 µm.

**Figure 9:**
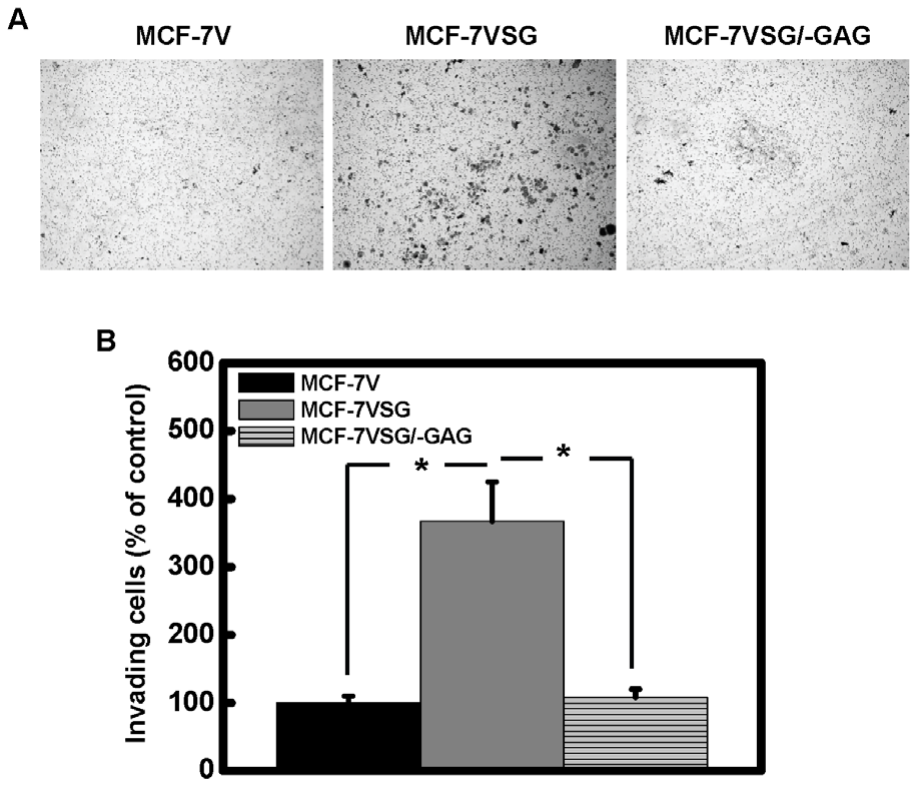
Serglycin promotes breast cancer cell invasion. 1×10^5^ cells were suspended in culture medium supplemented with 0.5% FBS and loaded onto the top of Transwell chambers equipped with matrigel-coated cell culture inserts. As chemotactic stimuli in the bottom chambers was used culture medium supplemented with 10% FBS. After 72 h of incubation, cells on the upper surface of the filter were mechanically removed with a cotton swab, and those which invaded underneath the surface were stained with Giemsa and counted. (A) Representative photos of cells invaded through matrigel and quantification (B). Data are given as means and SD of three independent experiments. Statistical significance of variances was calculated using a one-way ANOVA test. Asterisk (*) indicates statistically significant differences (*p*<0.05).

To evaluate the migratory capacity of breast cancer cells over-expressing serglycin, we performed a wound healing assay (Fig. 8A). We found that over-expression of intact serglycin (MCF-7VSG) markedly induced cell migration 24 h after wounding, and closure of the wound was achieved in 48 h (Fig. 8B). Mock-transfected MCF cells (MCF-7V) and MCF-7VSG/−GAG exhibited much lower migration 24 h post-wounding (approximately 50% less), and closure was not achieved at 48 h after wounding (Fig. 8A and B). We also measured the motility of cancer cells using a transwell assay. We found that MCF-7VSG cells expressing intact serglycin migrated significantly faster than mock transfected MCF-7V and MCF-7VSG/−GAG cells (Fig. 8C). In parallel wound healing assay experiments, MCF-7VSG cells that were at the edge of the wound at time 0 h as well as migratory cells at time 24 h were stained for serglycin and F-actin (Fig. 8D). Serglycin showed a cytoplasmic and pericellular membrane associated localization in MCF-7VSG grown at confluence and co-localized with the cortical F-actin on cell-cell adhesions (Fig. 8D, t = 0 h). In migratory cells, serglycin showed a filamentous cytoplasmic staining, but was also present in filopodia-like structures, co-localizing with actin (Fig. 8D, t = 24 h). The potential of cancer cells to invade through matrigel was also examined. Cells over-expressing serglycin (MCF-7VSG) exhibited significantly higher capability to invade through matrigel compared to mock transfected MCF-7V cells and cells expressing non-glycanated serglycin (MCF-7VSG/−GAG) (Fig. 9A and B).

## Discussion

Various studies have shown that the tumor cell microenvironment undergoes significant re-organization during tumorigenesis and is characterized by abnormal expression of PGs by cancer cells and accumulation of PG species in the tumor stroma [17], [18], [26], [27], [28]. The majority of matrix-accumulated PGs are synthesized by cancer activated stromal fibroblasts. It is well established that breast cancer cells do not synthesize significant amounts of matrix-secreted PGs, but mainly express cell-surface associated PGs, such as CSPG4, syndecans and glypicans, which promote tumor growth and spread [17], [29], [30], [31], [32]. We demonstrated for the first time that serglycin is expressed both at mRNA and protein levels in breast cancer cells and we found increased expression of this PG in aggressive breast cancer cells. More importantly, aggressive breast cancer MDA-MB-231 cells constitutively secreted significant amounts of serglycin into the culture medium. In this study, we isolated and characterized biochemically the PGs secreted in the culture medium, and demonstrated that serglycin represented the major PG population secreted by this cell line. Serglycin had an average molecular size of ∼300 kDa and carried exclusively CS side chains. The CS chains consisted mainly of 4-sulfated disaccharides (87%), with much less 6-sulfated (10%) and non-sulfated (3%) disaccharides. CS chains of serglycin secreted by breast cancer cells did not contain detectable amounts of oversulfated disaccharides [disulfated (4,6) disaccharides] found in serglycin from hematopoietic cells, such as basophils, monocytes, macrophages, connective tissue, and mucosal mast cells [1], [33]. The disaccharide composition was similar to that of CS chains attached on serglycin secreted by myeloma cells that also carried predominantly 4-sulfated disaccharides (up to 93%) [9].

In a recent study, we demonstrated that CS chains with specific structure present on serglycin isolated from myeloma cells inhibited the classical complement pathway through direct binding to C1q, whereas the overall structure of serglycin was involved in the binding of MBL and the inhibition of the lectin pathway [13]. In this study, we clearly demonstrated that serglycin secreted by MDA-MB-231 breast cancer cells exhibited a similar capacity to inhibit the classical and the lectin pathways via binding to C1q and MBL with slightly different affinities than myeloma-derived serglycin. The inhibitory activity of serglycin was specific and restricted to these two pathways and did not involve the alternative pathway. It has been demonstrated previously that complement components such as C1q, C3, C3a, C4, C5 and the MAC were deposited in the inflammatory tumor microenvironment. The assumption has been that these activated complement proteins play a role in tumor defense directly through complement-dependent cytotoxicity and indirectly through antibody-dependent cell-mediated cytotoxicity [15]. These pathways were activated by factors present on tumor cells or induced by treatment of tumor cells with therapeutic antibodies. The complement system can be activated by tumor-binding antibodies, immune complexes or as a consequence of apoptosis or proteolytic processes [34], [35], [36], [37]. The mechanism of complement activation in cancer is known to involve mainly the classical and also the lectin pathways [15], [16], [38]. Since C1q can bind to numerous targets, the factors that initiate complement activation remain to be determined. However, malignant cells are known to express a variety of complement inhibitors [15], including CD35, CD46, CD55, CD59, factor H, complement inhibitory factor I and C4b-binding protein, which all attenuate complement cytotoxicity [39], [40], [41], [42], [43]. Our data from this and our previous study [13] demonstrate a role of serglycin secreted by malignant cells as an inhibitor of complement activation in the tumor microenvironment that may protect tumor cells from complement attack in the early phase of disease or later during immunotherapy.

Importantly, we confirmed the overexpression of serglycin in breast cancer cells *in vivo* in patient material by immunohistochemistry. Although serglycin exhibited a moderate expression in normal mammary epithelial cells, it was found to be highly expressed in breast cancer cells in all tissues examined showing a strong cytoplasmic and cell-surface associated localization. Serglycin was first identified in the rat yolk sac carcinoma cell line L2 and in F9 teratocarcinoma cells [44]. Since then, its expression has been documented in numerous hematological malignancies [9], [45], [46], [47], [48], and has been proposed to be a selective biomarker for acute myeloid leukemia [49]. In a recent study, high-throughput gene expression profiling analysis of nasopharyngeal carcinoma cell lines revealed the upregulation of serglycin in cells with high-metastatic potential in comparison to cells showing low-metastatic potential [10]. The clinical data revealed a potent prognostic importance of serglycin, since it serves as an independent prognostic indicator for disease-free survival and distant metastasis-free survival of patients. Studies to investigate the prognostic importance of serglycin expression in breast cancer are in progress in our laboratory. We found high expression levels for serglycin in cancer tissues and a correlation of higher expression of serglycin with tumor grade in breast cancer (manuscript in preparation). This is in agreement with findings in nasopharyngeal cancer and correlates serglycin overexpression with aggressive cancer phenotype.

The promotion of an aggressive cancer cell phenotype by serglycin overexpression was confirmed by stable transfection of low aggressive MCF-7 cells with serglycin cDNA. MCF-7 cells that transfected with intact serglycin cDNA secreted to the culture medium significant amounts of highly glycanated and low glycanated serglycin. Upregulation of serglycin only slightly affected breast cancer cell proliferation in cells grown on plastic surfaces. Similarly, serglycin overexpression did not affect the proliferation of nasopharygeal cancer cells [10]. Silencing of serglycin in nasopharygeal cancer cells significantly inhibited cancer cell migration and invasion suggesting a critical role for serglycin in cancer cell spread [10]. Here, we noticed a marked stimulation of anchorage-independent growth, migration and invasion of breast cancer cells over-expressing glycanated serglycin. This stimulatory effect of serglycin in the promotion of cancer cell growth, migration and invasion was abolished by removing the GAG attachment sites and thus deletion of GAG chains from the serglycin molecule. The specific structure of CS-4 present on serglycin may be important for serglycin functions in breast cancer. Altered biosynthesis of CS chains has been demonstrated in various cancer types. Specific structures of CS influence various biological processes during tumor growth and spread [50]. CHST11 gene that specifically mediates 4-O sulfation of CS was highly expressed in MDA-MB-231 breast cancer cells and breast cancer tissues. CS-4 chains mediated the binding of breast cancer cells to P-selectin and facilitated the formation of metastasis [51]. A known PG that carries CS-4 chains in breast cancer cells was cell surface associated CSPG4 [51]. CSPG4 is also present in melanoma and promotes tumor invasion and metastasis. Free CS-4 chains or CS-4 chains on CSPG4 bound to proMMP-2 and MT3-MMP forming a complex that facilitated the activation of proMMP2 by MT3-MMP-expressing tumor cells to enhance invasion and metastasis [52]. Apart from CS-4 chains of serglycin that may bind to proteases its core protein also interacts with proMMP9 [12] and MMP13 [53] and this association may promote their activation and modulate their properties. The ability of cancer cells to become anchorage-independent and adapt to their new environment in sites of metastasis avoiding anoikis (cell-detachment-induced apoptosis) is a hallmark of cancer cells. Strategies adapted by cancer cells to overcome anoikis include epithelial to mesenchymal transition (EMT), altered expression of integrins, hyperactivation of growth factor receptors, autophagy and entosis [54]. In our study, overexpression of serglycin in MCF-7 cells promoted a more migratory and invasive cell phenotype with increased formation of stress fibers but without a significant induction of a mesenchymal phenotype (data not shown). Serglycin was highly expressed by aggressive nasopharyngeal cancer cells showing a mesenchymal phenotype with high expression levels of mesenchymal markers and low levels of epithelial marker E-cadherin [10]. Serglycin suppression in aggressive nasopharyngeal cells failed to revert to epithelial phenotype, but significantly reduced the expression of the mesenchymal marker vimentin [10]. Similarly, serglycin was inversely correlated with the expression levels of E-cadherin and positively correlated with the expression level of the mesenchymal marker vimentin in primary nasopharyngeal tumor tissues by immunohistochemistry. Our data have now showed that serglycin is highly expressed by aggressive MDA-MB-231 breast cancer cells, which belong to the Basal B subgroup, and they show mesenchymal phenotype, enhanced invasive properties and enriched expression of EMT transcriptional drivers [21]. These cells exhibit an EMT gene signature and are found to resemble breast cancer stem cells, being CD44^high^CD24^low^ [55]. CD44 is involved in cell-cell and cell-matrix interactions and signals through several pathways regulating cancer cell’s EMT, proliferation, migration and invasion [56]. Serglycin is a ligand for CD44 in hematopoietic cells [57]. Secreted serglycin may interact with CD44 on breast cancer cell membrane and trigger CD44 signaling promoting cancer cell migration and invasion. In MDA-MB-231 and MCF-7 cells transfected with intact serglycin cDNA, this PG exhibited a filamentous staining and localization in filopodia-like structures. The partial co-localization of serglycin with actin in cell-cell adhesions in non-migrating cells and filopodia-like structures in migratory cells, suggests a role for serglycin in the organization of the cytoskeleton or recycling/trafficking of functional molecules in cell membrane that participate in migration and invasion. This notion was further strengthened by our preliminary data that serglycin was co-precipitated with proteins involved in actin cytoskeleton re-organization during cell movement (not shown).

In conclusion, the data presented in this study demonstrated for the first time that serglycin is a major proteoglycan in breast cancer cells and is secreted into the culture medium, but also present in the cytoplasm in vesicles and at the plasma membrane. Our data expand our knowledge on serglycin biology, showing that serglycin promotes breast cancer cell migration, invasion and anchorage-independent growth in a CS-dependent manner and inhibits complement system activity in the tumor microenvironment.
